# Impact of Socio-Demographic Factors on Quality of Life and Coping Strategies of Children with Different Disabilities

**DOI:** 10.3390/medicina60101638

**Published:** 2024-10-07

**Authors:** Ayoob Lone, Abdul Sattar Khan, Fahad Abdullah Saeed AlWadani, Abdullah Almaqhawi

**Affiliations:** 1 Department of Clinical Neurosciences, College of Medicine, King Faisal University, Alhasa 31982, Saudi Arabia; mlone@kfu.edu.sa; 2 Department of Family Medicine, College of Medicine, King Faisal University, Alhasa 31982, Saudi Arabia; aalmughawi@kfu.edu.sa; 3 Department of Ophthalmology, College of Medicine, King Faisal University, Alhasa 31982, Saudi Arabia; falwadani@kfu.edu.sa

**Keywords:** quality of life, coping strategies, disability, children, Saudi Arabia

## Abstract

*Background and Objectives*: Children with disabilities face unique challenges that can affect their well-being and quality of life (QOL). This study aimed to assess the QOL and coping strategies adopted by children with disabilities and explore how socio-demographic factors influence QOL and coping strategies. *Materials and Methods*: This cross-sectional study, which was conducted in Saudi Arabia with children aged 6 to 18 years, used a stratified random sample to ensure representation from a variety of demographic groups. Short Form-12 (SF-12) was used to assess the QOL in the sample population. The Brief COPE Inventory was used to examine coping strategies among the children. One-way analysis of variance was applied to examine differences in the QOL, coping strategies scores, and demographic variables. Multiple regression analyses were performed to examine the role of demographic variables in predicting QOL and *p* value was considered statistical significance at *p* < 0.05. *Results*: The results of the study clearly revealed significant differences between the mean scores of QOL for gender, age, and type of disability, duration of disability, education qualification, family status, family occupation, and housing status. Female participants showed better QOL in physical functioning than their male counterparts. Children with intellectual disability reported better QOL in general health, vitality, social functioning, and mental health. Participants with seven to eight years of disability reported higher scores in physical functioning, vitality, and mental health. Children whose parents were working in private agencies and living in rented houses scored higher on the general health aspects of quality of life. The findings also revealed that the duration of the disability was a significant predictor of the QOL. The mean scores of different dimensions of coping strategies clearly revealed that male participants used dysfunctional coping (*p* < 0.01), as compared to problem-focused and emotional-focused coping while female children relied more on emotional-focused coping. Emotion-focused coping was significantly higher in participants with visual (*p* < 0.01), learning (*p* < 0.01), and intellectual disabilities (*p* < 0.01). Dysfunctional coping scores were higher among children with auditory disabilities (*p* < 0.01) and multiple disabilities (*p* < 0.01). *Conclusions*: This study highlights the significance of demographic factors in understanding and improving the well-being of a diverse population of disabled juveniles. It offers valuable insights into the subtle factors affecting quality of life. Future interventions and policies can leverage these findings to enhance the quality of life of individuals with disabilities and to foster a more supportive and inclusive approach.

## 1. Introduction

Understanding the prevalence and dynamics of disabilities among children is imperative in the realm of public health and social welfare, to foster an inclusive and supportive society. This will negatively affect the individual, family, and the entire society if the system does not support them and their quality of life (QOL) [1]. Saudi Arabia, a nation undergoing rapid socio-economic transformations, stands at the intersection of tradition and modernity, presenting a unique context for investigating the current status of disability among its children under the age of 18 years. With the global shift towards recognizing the rights of individuals with disabilities, it is becoming increasingly vital to investigate how Saudi Arabia, as a member of the international community, addresses the needs and challenges faced by children with disabilities.

Globally, the rights and needs of individuals with disabilities have gained increasing recognition. The International Classification of Functioning, Disability, and Health (ICF) framework and associated questions are being used by numerous countries in their national disability surveys and consensus-building efforts [2,3,4]. As per the recent analyses report, more than 1.3 billion (16%) people worldwide are experiencing some kind of disability [5]. According to the Global Burden of Disease analysis, 15.3% of the world population (978 million people out of an estimated 6.4 billion) had “moderate or severe disability”, [6] in comparison 2.9%, or approximately 185 million, had “severe disability” [7].

Based on data from a national demographic survey, more than half a million people, or one in every 30 people in Saudi Arabia, were estimated to have a disability in 2016. By 2021, this number had increased to 1.4 million [8]. There is currently a dearth of psychological research on the quality of life of people with different disabilities in Saudi Arabia. People with disabilities may require specialized medical, social, psychological, vocational, and other rehabilitation services [9,10,11]. Moreover, few studies have examined the prevalence and nature of disabilities among Saudi children. For example, research published in 2018 illustrated a survey conducted in 2016 as reported by the General Authority for Statistics, Saudi Arabia (N = 20,064,970) highlighting that a higher prevalence rate of disability was observed among men aged 60 years and above [8]. Moreover, males make up 52.2% of the population, and females make up 47.8%. Geographically, the survey results indicated that the Riyadh region has the highest disability rate (25.13%) compared to other Saudi Arabian regions [12]. Another study conducted in Riyadh, Saudi Arabia, reported a disability prevalence of 2.51% in children aged 2 to 4 years with a 3:1 female-to-male ratio [13]. Another study in Makkah and Jeddah found a prevalence of 2.62 to 3.68 per 1000 children for autism spectrum disorders [14].

This research was necessary because children with disabilities require specialized medical, psychological, and social services. In the Saudi context, there is a pressing need to understand the factors that influence their QOL and the coping strategies they use to manage their condition. The quality of life is a wide-ranging notion that is intricately influenced by an individual’s physical and mental well-being, degree of independence, social connections, personal convictions, and interactions with prominent aspects of their surroundings [15]. Long-term caregiving for children with chronic disabilities can be detrimental to their QOL.

Specifically, this study investigates the prevalence of disabilities among children in Saudi Arabia, assesses their QOL, and examines how socio-demographic factors such as age, gender, socioeconomic status, and family structure affect coping mechanisms. Several studies have proposed mediating factors in this regard, which can be grouped into the following categories: child characteristics, such as age, sex, type of disability, and behavior of the families of disabled children [16,17]. However, no study has assessed the influence of these factors on the QOL of disabled children so far. Given the lack of comprehensive data on QOL and coping mechanisms of children with disabilities in Saudi Arabia, this study fills a crucial gap in the literature.

The international context provides valuable comparisons, but the primary focus of this paper remains on Saudi Arabia. Globally, children with disabilities are known to use a wide array of behavioral, cognitive, and emotional coping methods to effectively manage stressors and improve their resilience. These strategies, including problem-solving techniques such as helping others, positive reframing, acceptance, and avoidance, are examples of these tactics [18,19]. The nature of the condition, individual variations, and contextual factors such as family support and societal views towards disability can affect the effectiveness of these tactics [20,21]. However, Saudi-specific studies that detail these coping methods and their effectiveness remain scarce.

The coping experiences of children with disabilities are greatly influenced by their socio-demographic characteristics globally. In Saudi Arabia, the socio-demographic factors such as, age, gender, family structure, race/ethnicity, and financial level might affect the cultural norms surrounding disabilities and the resources available to individuals [22,23]. Children from minority backgrounds may experience stigma and discrimination because of their disability, whereas children from low-income families may experience additional stressors related to financial constraints and limited access to healthcare services [24,25]. Identifying these challenges and understanding the unique needs of these groups is critical for creating equitable support systems.

While Saudi Arabia has made considerable progress in offering services to persons with disabilities, accessibility remains uneven. Improvements are needed in specialized care, gender-sensitive services, and the availability of trained professionals. Efforts to integrate persons with disabilities into mainstream education and employment are growing, but continued support from both the government and community is necessary for access that is more equitable to services. Additionally, the implementation of the “Saudi Vision 2030” plan has set ambitious goals for improving the quality of life for persons with disabilities, yet challenges in policy execution and regional disparities persist [26].

The primary objective of this study was to investigate how children with disabilities in Saudi Arabia manage the challenges associated with their conditions and to assess the impact of these coping strategies on their QOL. Nonetheless, understanding the ways in which these children cope with their disabilities is crucial for identifying factors that contribute to their overall well-being. In addition to this primary focus, the study also sought to examine the role of socio-demographic factors such as age, gender, socioeconomic status, and family structure in shaping coping mechanisms and influencing the QOL of children with disabilities. Furthermore, the study will inform the development of targeted interventions and support systems tailored to the needs of children with disabilities and their families, ultimately contributing to their improved well-being and integration into society.

## 2. Materials and Methods

### 2.1. Study Design

The present study consisted of a cross-sectional design sampling children with disabilities in Saudi Arabia. The research was conducted at a specific point in time in the Kingdom of Saudi Arabia, and the study depicted the setup and characteristics of disability. The desired modality enabled researchers to obtain data from multiple areas within the population, thus allowing them to achieve a global view of the issue. Ethical approval for the study was granted by the Deanship of Scientific Research at King Faisal University in AlHasa, Saudi Arabia (KFU-REC-2023-SEP-ETHICS1350), in compliance with the Helsinki Declaration on research involving human subjects. Every participant was informed about the purpose and objectives of the research, and the survey was conducted after all necessary ethical requirements were met.

### 2.2. Participants and Sampling

This study focused on children aged from 6 to 18 years residing in Saudi Arabia. A stratified random sampling method was employed to ensure that the sample was representative of the population across key demographics, including geographical regions, urban and rural settings, and socioeconomic strata. The population was first divided into distinct strata based on these factors like geographical region (eastern, western, northern, southern, and central), urban vs. rural and socioeconomic status (low, middle, and high) ensuring each group was proportionately represented in the final sample. A random sample was then selected from each of these strata to ensure comprehensive representation.

### 2.3. Sample Size Calculation

The total sample size of 415 was calculated using a formula (n = Z^2^ × p × (1 − p)/E^2^) designed for stratified random sampling and cross-sectional study design [8], which took into account the prevalence of disability among children in Saudi Arabia [13,14], a 95% confidence level, and a 5% margin of error. After calculating the sample size, adjustments were made to account for potential non-response and incomplete data, resulting in the final sample size of 415. However, only 369 participants completed the survey and were included in the analysis.

### 2.4. Data Collection Tools

To achieve the goal, the present study used various measures including Short Form-12 (SF-12) to assess the QOL. Brief COPE Inventory was used to evaluate the coping strategies. The study also included a demographic questionnaire prepared by the researchers.

Short-Form-12: Quality of life was assessed using Short Form-12 (SF-12). The SF-12 health survey was the SF-12v2, an abbreviated version of SF-36 [27]. The 12 items were shown to predict at least 90% of the physical and mental summary scales derived from SF-36 [28]. This self-reported scale measures eight domains: physical functioning, role physical, bodily pain, general health, vitality, social functioning, role emotional, and mental health. In the present study, the physical component scale (PCS-12) and mental component scale (MCS-12) consisted of six items each and were computed and normalized for the SF-12v2 according to published algorithms [29]. The scores ranged from zero to 100, with higher scores indicating better physical and mental health [30]. A score of ≤50 on the PCS-12 has been recommended as the cutoff to determine a physical condition, and a score of ≤42 on the MCS-12 indicated clinical depression [30]. The physical component summary and the mental component summary showed a good internal consistency reliability, as evidenced by alpha coefficients of 0.89 and 0.76, respectively [31]. The internal consistency reliability (Cronbach’s alpha) of the measure used in the present study was found to be 0.80 and 0.76, respectively.

Brief COPE: Coping strategies were evaluated using the brief coping inventory. Brief COPE is a 28-item scale designed to measure effective and ineffective coping strategies in response to stressful life events [32]. Brief COPE consists of 14 subscales, including self-distraction, active coping, denial, substance abuse, emotional support, use of information support, behavioral disengagement, venting, positive reframing, planning, humor, acceptance, religion, and self-blame. A 4-point Likert scale, with 1 representing “not at all” and 4 representing “a great deal”, was used by respondents to rate their use of coping mechanisms. The coping score for each subscale was calculated as the sum of the individual item scores. A high score on this scale indicates greater use of any specific coping strategy [33]. Based on Cooper’s categorization model, coping strategies were divided into three categories: problem-focused coping (active coping, using information support, and planning), emotion-focused coping (acceptance, humor, positive reframing, and emotional support), and dysfunctional coping (behavioral disengagement, denial, distraction, self-blame, substance abuse, and venting) [34]. According to Meyer’s model, coping can be divided into two categories such as adaptive coping (problem- and emotion-focused coping) and maladaptive coping (dysfunctional coping) [35]. As reported by Carver et al. (1989), the internal consistency reliability of the COPE inventory ranged from 0.42 to 0.89 [36]. In this study, the internal consistency reliability for this measure (Cronbach’s alpha) ranged from 0.43 to 0.85 in the current sample.

Demographic Questionnaire: The instrument covered demographic information such as age, gender, type and duration of disability, and educational experiences. Furthermore, details regarding their families such as living areas, family type, income, occupation, and housing status, were provided.

### 2.5. Procedure

Trained senior medical students and interviewers conducted face-to-face interviews with the parents or guardians of the selected children to ensure confidentiality and cultural sensitivity. Collaboration between healthcare facilities and educational institutions has facilitated access to medical and academic records. Informed consent was obtained from the parents or guardians prior to data collection, emphasizing voluntary participation. Measures were implemented to safeguard the participants’ privacy, including data anonymization and secure storage.

### 2.6. Statistical Analysis

Statistical Package for Social Sciences (SPSS) software (version 27.0; IBM, SPSS, Chicago, IL, USA) was used for statistical analysis. Descriptive statistics were used to characterize the study population and provide an overview of the prevalence and distribution of disabilities. Stratified analyses have explored variations in the prevalence of disabilities across different demographic and socioeconomic strata. We examined the statistical association between categorical variables using the chi-squared test. One-way analysis of variance was used to examine differences in the QOL, coping strategies scores and demographic variables. Multiple regression analyses were applied to examine the role of demographic variables in predicting QOL and *p* value was considered statistical significance at *p* < 0.05.

## 3. Results

Table 1 presents the demographic characteristics of the study participants. This study invited 415 children with different disabilities to be enrolled in various rehabilitation centers in the Alhasa region of Saudi Arabia. A total of 369 children (226 males and 143 females) completed the questionnaire. The remaining 46 participants, who were reluctant to respond to all items in the questionnaire, were excluded. Male participants had a significantly higher frequency of disability than did female participants (*p* = 0.00). Autism is the most common disability among male children. Respondents aged 6 to 12 years suffered significantly more (*p* = 0.00) than the other age groups. The findings also revealed a significantly higher frequency of various disabilities in patients with longer disability duration. Furthermore, compared to middle and high school students, participants in elementary classes had a significantly higher frequency of disability (*p* = 0.00). In addition, it was also observed that the percentage of disability was significantly higher in children belonging to nuclear families (*p* = 0.00) as compared to joint families. No significant differences were found between disabilities in terms of the area of residence, monthly income, family occupation, or housing status.

The mean scores of different domains of quality of life and SDs for different demographic variables presented in Table 2 clearly revealed significant differences between the mean scores of QOL for gender, age, type of disability, duration of disability, education qualification, family status, family occupation, and housing status. The mean scores of the different domains of quality of life clearly revealed that female participants showed better QOL in terms of physical functioning (M = 5.13, SD = 1.22, *p* = 0.01) than their male counterparts. In terms of the age, children aged 6 to 12 years had a better QOL for physical (M = 3.50, SD = 0.75, *p* = 0.05) and emotional roles (M = 3.46, SD = 0.79, *p* = 0.05), whereas those aged 13 to 18 years scored higher in bodily pain (M = 2.35, SD = 1.35, *p* = 0.05) and mental health (M = 6.71, SD = 1.96, *p* = 0.05). With regard to the type of disability, children with intellectual disability reported better QOL in terms of general health (M = 2.94, SD = 0.74, *p* = 0.01), vitality (M = 4.76, SD = 7.38, *p* = 0.01), social functioning (M = 2.82, SD = 1.50, *p* = 0.01), and mental health (M = 6.88, SD = 1.86, *p* = 0.01). Children with hearing problems had better QOL in physical functioning (M = 5.28, SD = 1.20, *p* = 0.01), role physical (M = 3.69, SD = 0.66, *p* = 0.01), and role-emotional (M = 3.79, SD = 0.52, *p* = 0.01). For duration of disability, children with seven to eight years of disability perceived a higher score in physical functioning (M = 5.15, SD = 1.18, *p* = 0.05), vitality (M = 4.00, SD = 1.50, *p* = 0.01), and mental health (M = 6.90, SD = 2.03, *p* = 0.01). Participants with nine to ten years of disability reported higher scores for role physical (M = 3.74, SD = 0.69, *p* = 0.01) and role-emotional (M = 3.71, SD = 0.60, *p* = 0.01). Bodily pain (M = 2.42, SD = 1.37, *p* = 0.01) and social functioning (M = 2.48, SD = 1.59, *p* = 0.01) were better in children with a disability duration of >11 years.

Participants enrolled in elementary schools reported higher scores in physical functioning (M = 4.92, SD = 1.32, *p* = 0.01), role physical (M = 3.58, SD = 0.72, *p* = 0.01), and role-emotional (M = 3.54, SD = 0.76, *p* = 0.01). Only the general health dimension of QOL (M = 2.73, SD = 1.16, *p* = 0.01) was better among the children enrolled in middle schools. Participants enrolled in high school showed a better QOL in terms of social functioning (M = 2.41, SD = 1.62, *p* = 0.01) and mental health (M = 6.97, SD = 1.73, *p* = 0.01). In terms of family status, children belonging to joint families perceived higher scores for physical functioning (M = 5.02, SD = 1.35, *p* = 0.01), role physical (M = 3.55, SD = 0.76, *p* = 0.01), and role emotional (M = 3.53, SD = 0.76, *p* = 0.01), whereas children living in nuclear families reported higher scores for social functioning (M = 2.41, SD = 1.62, *p* = 0.05) and mental health aspects of QOL (M = 6.97, SD = 1.73, *p* = 0.05). It was also observed (Table 2) that children whose parents were working in private agencies (M = 2.85, SD = 1.20, *p* = 0.01) and living in rented houses (M = 2.68, SD = 1.10, *p* = 0.01) scored higher on the general health aspects of QOL.

The mean scores of the different dimensions of coping strategies and SDs for different demographic variables presented in Table 3 clearly revealed significant differences between the mean scores for sex, type of disability, family status, and area of residence. The mean scores of different dimensions of coping strategies clearly revealed that male participants used dysfunctional coping (*p* < 0.01), as compared to problem-focused and emotional-focused coping (*p* < 0.05 and *p* < 0.01, respectively), while female children relied more on emotional-focused coping than problem-focused and dysfunctional coping. Regarding the types of disabilities, emotion-focused coping was significantly higher in participants with visual (*p* < 0.01), learning (*p* < 0.01), and intellectual disabilities (*p* < 0.01). Dysfunctional coping scores were higher among children with auditory disabilities (*p* < 0.01) and multiple disabilities (*p* < 0.01). However, problem-focused coping was significantly higher in patients with autism spectrum disorder than in those with emotion-focused and dysfunctional coping.

In terms of family status, emotion-focused coping scores were higher in children belonging to joint and nuclear families (*p* < 0.01) than in problem-focused and dysfunctional coping (*p* < 0.01, and *p* < 0.01, respectively). It was also observed (Table 3) that children residing in urban areas (*p* < 0.05) showed higher scores on emotion-focused coping than on other coping strategies. Children living in rural areas reported higher scores on dysfunctional coping (*p* < 0.05) than on problem-focused (*p* < 0.01) and emotion-focused coping (*p* < 0.05).

Multiple regression analysis was applied to examine the role of demographic characteristics in predicting quality of life based on demographic selected variables shown in Table 4. The results presented in Table 4 revealed a significant contribution of ten predictor variables (i.e., gender, age, duration and type of disability, educational qualification, family status, area of residence, monthly income, family occupation, and housing status) in explaining scores on quality of life (R = 0.26, R^2^ = 0.07, F(10, 358) = 2.56, *p* < 0.01). These variables jointly explained 7% of the variance in the scores on quality of life. The regression coefficients indicated that the duration of disability was positively and significantly related to QOL (β = 0.13, t = 2.28, *p* < 0.01). This indicates that children with a long disability experience a better QOL than those with a short disability. None of the other demographic variables were found to be significant predictors of QOL among participants.

## 4. Discussion

People with disabilities comprise 16 percent (1.3 billion) of the world’s population, with 80 percent of people with different disabilities living in the Global South [5]. An estimation of 266 million children aged 0 to 19 years old experience moderate-to-severe disabilities worldwide [37]. This number is increasing as non-communicable diseases become more common and people live longer lives. People with disabilities comprise a diverse group, and factors such as gender, age, sexual orientation, religion, race, ethnicity, and socioeconomic status can influence their life experiences and health needs. People with different types of disabilities have poor health, short life spans, and experience more limitations in everyday functioning than those without disabilities [38,39]. The present study was conducted to examine the role of demographic factors that potentially influence the QOL and coping strategies of delinquents with different disabilities. There is currently a dearth of psychological research on the QOL and coping methods of children with disabilities in Saudi Arabia; however, many studies have reported only the QOL of caregivers of people with disabilities in the Saudi population [40,41,42,43,44]. This work will improve the QOL and explore effective coping strategies for people with disabilities and serve as a reliable source for future research.

### 4.1. Quality of Life and Demographic Characteristics

The results of the study reported that the physical functioning aspect of the QOL of disabled children was higher among female participants than among male participants. These results are inconsistent with previous research [45], which suggests that males have more opportunities to interact with society and the freedom to engage in social activities and social events. Thus, males reach an acceptable level of quality of life compared with females. The quality of life of disabled males compared to disabled females can vary based on various factors, such as access to healthcare, social support, and individual circumstances. One potential reason for disparities in quality of life could be related to social and cultural factors as well as differences in overall health and well-being. Additionally, studies have shown that disabled males may face challenges related to societal expectations of masculinity and self-reliance, which could impact their perceived quality of life.

Age also appears to be an important factor in some SF-12 domains. The results demonstrated that children aged between 6 and 12 years reported a better quality of life in terms of physical and emotional roles. Previous studies have described significant differences in the Child Health Questionnaire subscales of physical functioning, role functioning, parental impact and family activities, pain, and general health in disabled children [46]. However, children aged 13 to 18 years scored higher in bodily pain and mental health. In contrast, a study conducted by Linden-Bostrom and Persson (2015) reported that youth (13 to 18 years old) with multiple impairments had a higher probability of developing poor mental health [47].

There was a significant difference in the mean scores of SF-12 juveniles with different types of disabilities. These results are consistent with previous studies [48]. Specifically, children with intellectual disability reported better quality of life in terms of general health, vitality, social functioning, and mental health, while children with hearing problems showed better quality of life in physical functioning, physical role, and emotional role. Previous studies have shown that better health positively influences of quality of life of disabled people [49,50].

Some initial trends were observed across the duration of disabilities, where seven scales of the SF-12 showed a trend or an association with the duration of disabilities. The results demonstrated that children aged seven to eight years with disability perceived higher scores in physical functioning, vitality, and mental health. Participants with nine to ten years of disability reported higher physical and emotional role scores. Bodily pain and social functioning were higher in children whose disability duration was >11 years. Our results were inconsistent with those of previous studies, which reported that the QOL of people who were recently physically disabled may be affected more than those with a long duration of disability [51].

Different results were found when comparing the levels of education across different dimensions of QOL. Children enrolled in elementary schools reported better physical functioning and physical and emotional roles. Only the general health dimension of QOL was found to be better for children enrolled in middle school. The importance of education for people with disabilities significantly influences their quality of life, aligning with findings from previous studies [52,53]. Education provides individuals with essential skills and knowledge, enhancing their employment opportunities and independence. Moreover, inclusive educational environments that foster social interactions help build self-esteem, and promote personal development. These factors contribute to a greater sense of belonging and improved mental health, which are crucial for the overall quality of life.

Consistent with previous findings examining the impact of disease duration and disability [51,54,55], it was hypothesized that demographic variables can predict the QOL of children with disabilities. Our results indicated that only long duration of disability was positively and significantly associated with the QOL of disabled children. Children and their families often adapt to the challenges posed by a long-term disability over time. This process can foster resilience and coping strategies, allowing them to develop a positive outlook and better manage their circumstances. The familiarity with their situation may lead to more effective coping mechanisms and emotional regulation. Moreover, lengthier exposure to disability often means that children have had more time to develop skills tailored to their individual needs. They might benefit from therapies, adaptive technologies, and educational interventions that enhance their functionality and independence, contributing to a better quality of life. Furthermore, Children who have lived with a disability longer may have greater self-acceptance and understanding of their identity. This can lead to improved self-esteem and a more profound sense of belonging, thus positively impacting their overall well-being [56]. In Saudi Arabia, government initiatives and policies aimed at improving the welfare of disabled individuals, such as employment quotas, financial assistance programs, and accessibility regulations, may contribute to the overall well-being of disabled people. Strong family and community support networks are integral to Saudi society, providing emotional, practical, and financial assistance to individuals with disabilities [26,57]. However, caution should be exercised when generalizing the relationship between duration of disability and QOL. While in some cases, people with long duration of illness may develop coping mechanisms and find ways to adapt to their condition leading to a relatively good quality of life, this is not always the case. For many people, chronic illness can significantly impact physical, mental, and social well-being, leading to a decrease in QOL.

### 4.2. Coping Strategies and Demographic Characteristics

The findings of this study revealed that participants used emotion-focused coping and dysfunctional coping more than problem-focused coping. The results showed significant differences in coping strategies in terms of sex, type of disability, family status, and area of residence. Regarding gender, male participants used dysfunctional coping, whereas female children relied on emotion-focused coping. Although no study has directly compared our findings, previous research on normal samples revealed that females expressed more emotions and used more social support to manage stress, whereas, males may rely more on passive or maladaptive coping [58,59]. Several factors contribute to the perception that males might use more passive and maladaptive coping strategies in certain situations. Traditional gender roles often encourage males to suppress emotions and deal with problems independently, which can lead to the use of passive coping mechanism like avoidance and denial. Moreover, in certain cultures, men are expected to be strong and stoic, which can discourage them from seeking help to express vulnerability, resulting in the adoption of maladaptive coping strategies. In addition, males may face greater stigma when seeking help for mental health issues, leading them to resort to passive coping mechanisms rather than professional support. Overall, societal norms and expectations can influence males to use more passive coping strategies as they navigate through challenges and stressors. However, it is necessary to note that coping strategies vary greatly among individuals and are influenced by a variety of factors beyond gender. Previous research has reported that females tend to use more emotional-focused coping strategies, such as social support and religious coping, compared to males [60]. Certain factors contribute to the use of emotional-focused coping among females, such as socialization [61], biological difference [62], and culture [63]. However, it is important to recognize that individual differences exist, and that coping strategies can vary based on the specific context and personal characteristics of individuals.

Emotion-focused coping scores were significantly higher among children with visual, learning, and intellectual disabilities. Our results were consistent with those of previous studies, which reported that emotion-focused coping was the most effective coping strategy compared to problem-focused and dysfunctional coping among physically disabled people [64]. Our results also showed that children with auditory and multiple disabilities relied on dysfunctional coping strategies. Presently, there are no similar studies for comparison; however, Hemati et al. (2019) reported that mothers of children with hearing and visual impairments tended to use dysfunctional coping strategies [65]. In our study, participants with autism used problem-focused coping strategies. Unfortunately, there are no data available for the direct comparison of our findings. Wang et al. (2011) reported that parents of children with autism tend to use planning as a coping strategy more than parents of children with mental retardation [66].

Regarding the family status of the participants, emotional-focused coping scores were higher in children belonging to joint and nuclear families as compared to problem-focused and dysfunctional coping. Previous findings have reported that parents of children with cancer belonging to joint/extended families more often used escape-avoidance coping compared to those belonging to nuclear families [67]. Emotion-focused coping strategies can play a vital role in helping children with disabilities and their families navigate challenges. In nuclear families, close-knit structures provide a strong support system for implementing emotion-focused coping. The presence of extended family members in joint families may offer additional support and understanding.

Another significant factor was found in this study, children residing in urban areas showed higher mean scores on emotion-focused coping than on other coping strategies. Our results are partially in line with those of previous studies, which reported that people living in urban areas had higher emotion-focused and avoidant coping strategies than rural residents [68]. Previous findings have indicated that individuals living in urban areas are more inclined to use emotion-focused coping as a way of coping with stress and maintaining psychological well-being. A study conducted by Smith et al. (2017) found that residents of urban areas are more likely to engage in emotional expression and seek social support as a coping mechanism in response to daily stressors [69]. In our study, children living in rural areas reported higher scores on dysfunctional coping than on problem- and emotion-focused coping. Living in rural areas presents unique challenges for individuals seeking healthcare and coping with their medical conditions. Limited access to health care facilities, shortage of mental health professionals, and lack of resources may contribute to the use of dysfunctional coping strategies among patients in rural areas.

This study has some limitations. First, the study used a stratified random sample technique; selection bias may still be present, or variability within some strata may not be adequately captured. As a result, the findings might not comprehensively reflect the entire population, leading to biased results, especially if certain subgroups are under or overrepresented. Second, the study used self-reported data from parents and guardians, which raises the possibility of recall bias and the subjective interpretation of disability-related data. This subjective nature of the data can skew the results, affecting the overall prevalence rates and potentially leading to inaccurate conclusions regarding the extent of disabilities. Third, the study’s primary focus was on Saudi Arabian children, so extra care should be taken when extrapolating the results to other demographics or cultural settings. Cultural factors and different demographic settings might impact how disabilities are understood, reported, and managed. If applied to different contexts, the results may not hold the same significance, which limits their external validity. Finally, the study’s cross-sectional design means it captured data at a single point in time, preventing an analysis of the long-term effects of disabilities on the quality of life and other outcomes. A longitudinal study design is necessary to fully assess the long-term impact of disabilities on quality of life and other outcomes.

## 5. Conclusions

This study highlights key socio-demographic factors such as gender, age, type and duration of disability, and educational level as essential components in understanding and improving the QOL of children with disabilities. The findings revealed distinct coping patterns, with females more inclined to use emotion-focused strategies and males showing a tendency toward dysfunctional coping. Moreover, individuals with auditory and multiple disabilities often exhibit dysfunctional coping, whereas those with visual, learning, or cerebral disabilities are more likely to adopt emotion-focused coping mechanisms. These insights deepen our understanding of how different factors shape the quality of life and coping strategies of children with disabilities. The study’s contribution lies in identifying these interesting relationships, which can inform and lead the targeted interventions and policies aimed at enhancing coping mechanisms and overall quality of life for this vulnerable population. Future large-scale research should look at how coping mechanisms, community initiatives, and customized support systems can be put into place in order to help children with disabilities become more resilient and inclusive and, ultimately, to contribute to a more accepting and inclusive society.

## Figures and Tables

**Table 1:** General characteristics of the participants in the study (*n* = 369).

		Type of Disability						
	N (%) (*n* = 369)	Visual (*n* = 35)	Learning Disability (*n* = 97)	Autism (*n* = 125)	Intellectual Disability (*n* = 17)	Hearing (*n* = 73)	Mixed (*n* = 22)	*p* Value
Gender								0.01
Male	226	22	63	97	13	30	1	
Female	143	13	34	28	4	43	21	
Age								0.01
6–12 years	200	6	47	87	1	43	16	
13–18 years	169	29	50	38	16	30	6	
Duration of disability								0.01
1–2 years	39	1	16	16	0	6	0	
3–4 years	25	2	10	12	1	0	0	
5–6 years	40	4	13	14	0	9	0	
7–8 years	53	4	9	23	0	15	2	
9–10 years	63	5	18	17	1	14	8	
>11 years	149	19	31	43	15	29	12	
Education qualification								0.01
Elementary	156	3	29	68	1	39	16	
Middle	103	16	27	39	3	17	1	
High	110	16	41	18	13	17	5	
Family status								0.01
Joint	171	2	30	56	9	58	16	
Nuclear	198	33	67	69	8	15	6	
Area of residence								0.73
Urban	343	31	91	115	17	68	21	
Rural	26	4	6	10	0	5	1	
Monthly Income								0.74
<10,000 SAR	224	19	61	70	9	48	17	
10,001–15,000 SAR	116	13	29	44	6	19	5	
>15,000 SAR	29	3	7	11	2	6	0	
Family occupation								0.95
Government employees	191	14	51	55	13	45	13	
Private employee	83	8	25	36	2	11	1	
Business	87	13	20	30	2	15	7	
Others	8	0	1	4	0	2	1	
Housing status								0.90
Own	222	21	69	64	11	45	12	
Rented	147	14	28	61	6	28	10	

*p* < 0.01; *p* < 0.05.

**Table 2:** Quality of life of disabled people (mean and standard deviation) according to demographic factors (n = 369).

Factors	PF M ± SD	RP M ± SD	BP M ± SD	GH M ± SD	VT M ± SD	SF M ± SD	RE M ± SD	MH M ± SD
Gender								
Male	4.52 ± 1.45	3.38 ± 0.82	2.23 ±1.23	2.52 ± 1.17	3.90 ±2.44	2.31 ± 1.39	3.33 ± 0.82	6.57 ±2.07
Female	5.13 ± 1.22 **	3.47 ± 0.79	2.16 ± 1.29	2.39 ± 1.09	3.71 ± 1.53	2.06 ± 1.30	3.47 ± 0.82	6.37 ± 0.87
Age								
6–12 years	4.76 ±1.37	3.50 ± 0.75 *	2.07 ± 1.15	2.37 ±1.14	3.82 ± 1.48	2.16 ± 1.25	3.46 ± 0.79 *	6.31 ± 2.01
13–18 years	4.75 ± 1.43	3.31 ± 0.86	2.35 ± 1.35 *	2.59 ± 1.13	3.84 ± 2.71	2.28 ± 1.47	3.30 ± 0.85	6.71 ±1.96 *
Type of Disability								
Visual	4.65 ± 1.21	3.02 ± 0.95	1.97 ± 1.22	2.85 ± 1.03	3.82 ± 1.48	1.94 ± 1.25	3.05 ± 0.93	6.28 ± 1.38
Learning disability	4.74 ± 1.48	3.30 ± 0.83	2.19 ± 1.34	2.48 ± 1.36	3.78 ± 1.51	2.31 ± 1.48	3.22 ± 0.87	7.14 ± 2.35
Autism	4.42 ± 1.43	3.48 ± 0.75	2.30 ± 1.16	2.61 ± 1.04	3.48 ± 1.43	2.52 ± 1.35	3.32 ± 0.84	6.16 ± 1.85
Intellectual disability	5.11 ± 1.26	3.23 ± 0.90	2.64 ± 0.99	2.94 ± 0.74 **	4.76 ± 7.38 **	2.82 ± 1.50 **	3.35 ± 0.78	6.88 ± 1.86 **
Hearing	5.28 ± 1.20 **	3.69 ± 0.66 **	2.01 ± 1.34	1.91 ± 0.99	4.52 ± 1.40	1.64 ± 1.04	3.79 ± 0.52 **	6.73 ± 1.61
Mixed	4.75 ± 1.39	3.40 ± 0.85	2.31 ± 1.21	2.45 ± 0.80	3.04 ± 1.17	1.90 ± 1.01	3.68 ± 0.71	4.72 ± 1.75
Duration of disability								
1–2 years	4.76 ± 1.06	2.92 ± 0.87	2.33 ± 1.01	3.05 ± 1.12	3.35 ± 1.18	2.43 ± 1.07	2.97 ± 0.87	5.64 ± 2.00
3–4 years	4.72 ±1.40	3.36 ± 0.75	2.24 ± 1.09	3.00 ± 1.25	3.77 ± 1.53	2.24 ± 1.16	3.24 ± 0.83	5.76 ± 2.25
5–6 years	5.07 ± 1.20	3.50 ± 0.75	1.87 ± 1.13	2.42 ± 1.10	3.52 ± 1.66	2.10 ± 1.37	3.45 ± 0.81	6.41 ± 1.84
7–8 years	5.15 ± 1.18 *	3.56 ± 0.74	2.15 ± 1.30	2.26 ± 1.12	4.00 ± 1.50 **	1.98 ± 1.08	3.50 ± 0.79	6.90 ± 2.03 **
9–10 years	4.84 ± 1.55	3.74 ± 0.59 **	1.82 ± 1.08	2.14 ± 1.02	3.93 ± 1.54	1.73 ± 0.98	3.71 ± 0.60 **	6.31 ± 1.79
>11 years	4.50 ± 1.48	3.34 ± 0.85	2.42 ± 1.37 **	2.45 ± 1.11	3.98 ± 2.81	2.48 ± 1.59 **	3.32 ± 0.85	6.83 ± 1.98
Education qualification								
Elementary	4.93 ± 1.32 **	3.58 ± 0.72 **	2.05 ± 1.19	2.27 ± 1.06	3.81 ± 1.53	1.98 ± 1.14	3.54 ± 0.76 **	6.08 ± 1.87
Middle	4.39 ± 1.40	3.22 ± 0.85	2.23 ± 1.16	2.73 ± 1.16 **	3.69 ± 1.46	2.36 ± 1.29	3.24 ± 0.85	6.60 ± 2.30
High	4.84 ± 1.44	3.37 ± 0.84	2.39 ± 1.40	2.50 ± 1.17	3.98 ± 3.15	2.41 ± 1.62 **	3.30 ± 0.85	6.97 ± 1.73 **
Family status								
Joint	5.02 ± 1.35 **	3.55 ± 0.76 **	2.16 ± 1.25	2.36 ± 1.07	3.76 ± 1.53	2.07 ± 1.27	3.53 ± 0.76 **	6.27 ± 1.94
Nuclear	4.52 ± 1.39	3.30 ± 0.83	2.23 ± 1.26	2.56 ± 1.18	3.89 ± 2.54	2.34 ± 1.41 *	3.26 ± 0.85	6.68 ± 2.02 *
Area of residence								
Urban	4.77 ± 1.38	3.42 ± 0.80	2.18 ± 1.22	2.46 ± 1.14	3.58 ± 2.15	2.19 ± 1.35	3.39 ± 0.82	6.49 ± 1.90
Rural	4.53 ± 1.50	3.30 ± 0.92	2.46 ± 1.58	2.57 ± 1.06	3.53 ± 1.79	2.50 ± 1.42	3.30 ± 0.83	6.46 ± 2.96
Monthly Income								
<10,000 SAR	4.83 ± 1.39	3.50 ± 0.76	2.21 ± 1.21	2.53 ± 1.17	3.96 ± 2.44	2.19 ± 1.31	3.43 ±0.80	6.58 ± 2.16
10,001–15,000 SAR	4.57 ± 1.38	3.28 ± 0.87	2.22 ± 1.33	2.45 ± 1.11	3.54 ± 1.49	2.25 ± 1.47	3.31 ± 0.86	6.31 ± 1.68
>15,000 SAR	4.89 ± 1.42	3.34 ± 0.85	2.03 ± 1.26	2.06 ± 0.92	3.96 ± 1.61	2.27 ± 1.27	3.34 ± 0.85	6.51 ± 1.76
Family occupation								
Government employees	4.74 ± 1.42	3.37 ± 0.85	2.25 ± 1.33	2.27 ± 1.07	3.87 ± 2.58	2.07 ± 1.25	3.41 ± 0.82	6.56 ± 1.82
Private employee	4.86 ± 1.22	3.36 ± 0.80	2.20 ± 1.15	2.85 ± 1.20 **	3.80 ± 1.47	2.42 ± 1.53	3.21 ± 0.87	6.28 ± 2.04
Business	4.59 ± 1.50	3.52 ± 0.72	2.10 ± 1.16	2.56 ± 1.14	3.75 ± 1.52	2.33 ± 1.36	3.45 ± 0.78	6.56 ± 2.33
Others	5.75 ± 0.70	4.00 ± 0.00	2.12 ± 1.45	2.12 ± 0.99	3.75 ± 1.75	2.37 ± 1.76	3.75 ± 0.70	6.37 ± 1.50
Housing status								
Own	4.68 ± 1.42	3.39 ± 0.83	2.18 ± 1.27	2.32 ± 1.13	3.99 ± 2.47	2.16 ± 1.35	3.36 ± 0.84	6.61 ± 1.89
Rented	4.86 ± 1.34	3.46 ± 0.77	2.22 ± 1.22	2.68 ± 1.10 **	3.58 ± 1.43	2.30 ± 1.36	3.42 ± 0.80	6.31 ± 2.13

Note: PF = Physical Functioning; RP = Role-Physical; BP = Bodily Pain; GH = General Health; VT = Energy/Vitality; SF = Social Functioning; RE = Role-Emotional; MH = Mental Health; ** *p* < 0.01; * *p* < 0.05.

**Table 3:** Distribution of different coping strategies of disabled people (mean and standard deviation) across demographics (n = 369).

	Adaptive Coping						Maladaptive Coping		
	Problem-Focused Coping			Emotional-Focused Coping			Dysfunctional Coping		
Factors	Mean	SD	Sig.	Mean	SD	Sig.	Mean	SD	Sig.
Gender									
Male	15.10	4.91	0.02 *	24.60	7.42	0.00 **	24.72	8.77	0.00 **
Female	13.83	4.79		22.26	7.69		21.09	6.85	
Age									
6–12 years	14.53	5.17	0.72	23.23	8.03	0.14	23.87	9.05	0.11
13–18 years	14.70	4.57		24.40	7.06		22.48	7.14	
Type of Disability									
Visual Problem	15.08	4.15		26.71	6.43		21.20	4.84	
Learning disability	13.81	4.73		23.36	7.99		23.18	7.84	
Autism Spectrum disorder	24.15	7.75	0.01 **	15.32	4.83	0.01 **	23.17	7.71	0.01 **
Intellectual disability	13.70	4.48		23.58	7.02		23.41	8.08	
Auditory problem	15.63	5.16		24.64	7.93		25.10	10.49	
Multiple disability	10.63	4.28		15.95	5.83		17.77	6.57	
Duration of disability									
1–2 years	13.89	4.50		23.20	7.17		25.35	8.47	
3–4 years	14.24	4.48		22.92	7.30		24.72	7.02	
5–6 years	16.30	3.82	0.28	25.62	6.47	0.67	24.40	6.95	0.19
7–8 years	14.67	5.72		23.90	9.47		23.64	10.46	
9–10 years	14.15	5.71		23.26	7.96		21.60	8.53	
>11 years	14.57	4.60		23.72	7.21		22.67	7.60	
Educational level									
Elementary	14.28	5.45		22.69	8.40		23.46	9.20	
Middle	15.11	4.40	0.41	24.58	6.98	0.07	23.87	7.78	0.36
High	14.61	4.48		24.53	6.84		22.32	7.18	
Family status									
Joint	13.78	4.74	0.01 **	22.05	7.45	0.01 **	21.42	7.23	0.01 **
Nuclear	15.32	4.92		25.24	7.45		24.80	8.76	
Area of residence									
Urban	14.42	4.91	0.01 **	23.60	7.64	0.02*	22.96	8.23	0.02 *
Rural	17.07	3.99		25.88	7.02		26.80	7.84	
Monthly Income									
<10,000 SAR	14.58	5.09		23.50	7.82		23.58	8.80	
10,001–15,000 SAR	14.62	4.52	0.97	24.12	7.02	0.70	23.18	7.41	0.23
>15,000 SAR	14.82	5.00		24.37	8.44		20.79	6.67	
Family occupation									
Government employees	14.38	5.07		23.65	7.85		22.90	8.51	
Private employee	14.95	5.02	0.22	23.83	7.67	0.68	23.78	8.33	0.75
Business	14.49	4.48		23.65	7.35		23.26	7.65	
Others	17.87	1.95		27.00	2.67		25.37	8.17	
Housing status									
Own	14.50	4.94	0.62	24.16	7.65	0.22	23.01	8.17	0.52
Rented	14.76	4.84		23.17	7.53		23.57	8.38	

** *p* < 0.01; * *p* < 0.05.

**Table 4:** Result of multiple regression analysis predicting quality of life from demographic variables.

Predictors	b	SE-b	β	t	95% CI
Gender	0.08	0.08	0.54	0.98	−0.08–0.24
Age	−0.05	0.13	−0.04	−0.41	−0.30–0.20
Type of Disability	0.05	0.03	0.11	1.71	−0.01–0.12
Duration of disability	0.06	0.03	0.13	2.28 *	0.01–0.11
Education qualification	−0.06	0.08	−0.07	−0.79	−0.21–0.09
Family status	−0.09	0.08	−0.06	−1.04	−0.25–0.08
Area of residence	−0.14	0.15	−0.05	−0.97	−0.43–0.15
Monthly income	−0.04	0.06	−0.03	−0.59	−0.15–0.08
Family occupation	0.05	0.04	0.07	1.24	−0.03–0.14
Housing status	0.04	0.08	0.03	0.53	−0.11–0.20
R = 0.26					
R^2^ = 0.07					
F(10, 358) = 2.56 **					

** *p* < 0.01; * *p* < 0.05.

## Data Availability

The data that support our findings can be found by directly asking the corresponding author.
